# Biomedical Silicones: Leveraging Additive Strategies to Propel Modern Utility

**DOI:** 10.1021/acsmacrolett.2c00701

**Published:** 2023-01-20

**Authors:** Alec C. Marmo, Melissa A. Grunlan

**Affiliations:** † Department of Materials Science and Engineering Texas A&M University, College Station, Texas 77843-3003, United States; ‡ Department of Biomedical Engineering, Department of Materials Science and Engineering, Department of Chemistry Texas A&M University, College Station, Texas 77843-3003, United States

## Abstract

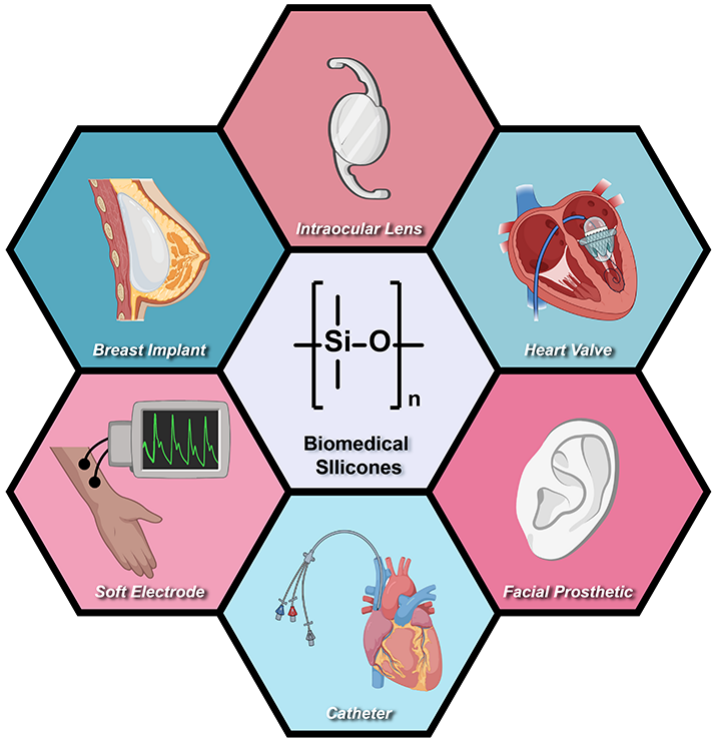

Silicones have a long history of use in biomedical devices, with unique properties stemming from the siloxane (Si–O–Si) backbone that feature a high degree of flexibility and chemical stability. However, surface, rheological, mechanical, and electrical properties of silicones can limit their utility. Successful modification of silicones to address these limitations could lead to superior and new biomedical devices. Toward improving such properties, recent additive strategies have been leveraged to modify biomedical silicones and are highlighted herein.

## 1 Introduction

**Figure 1:**
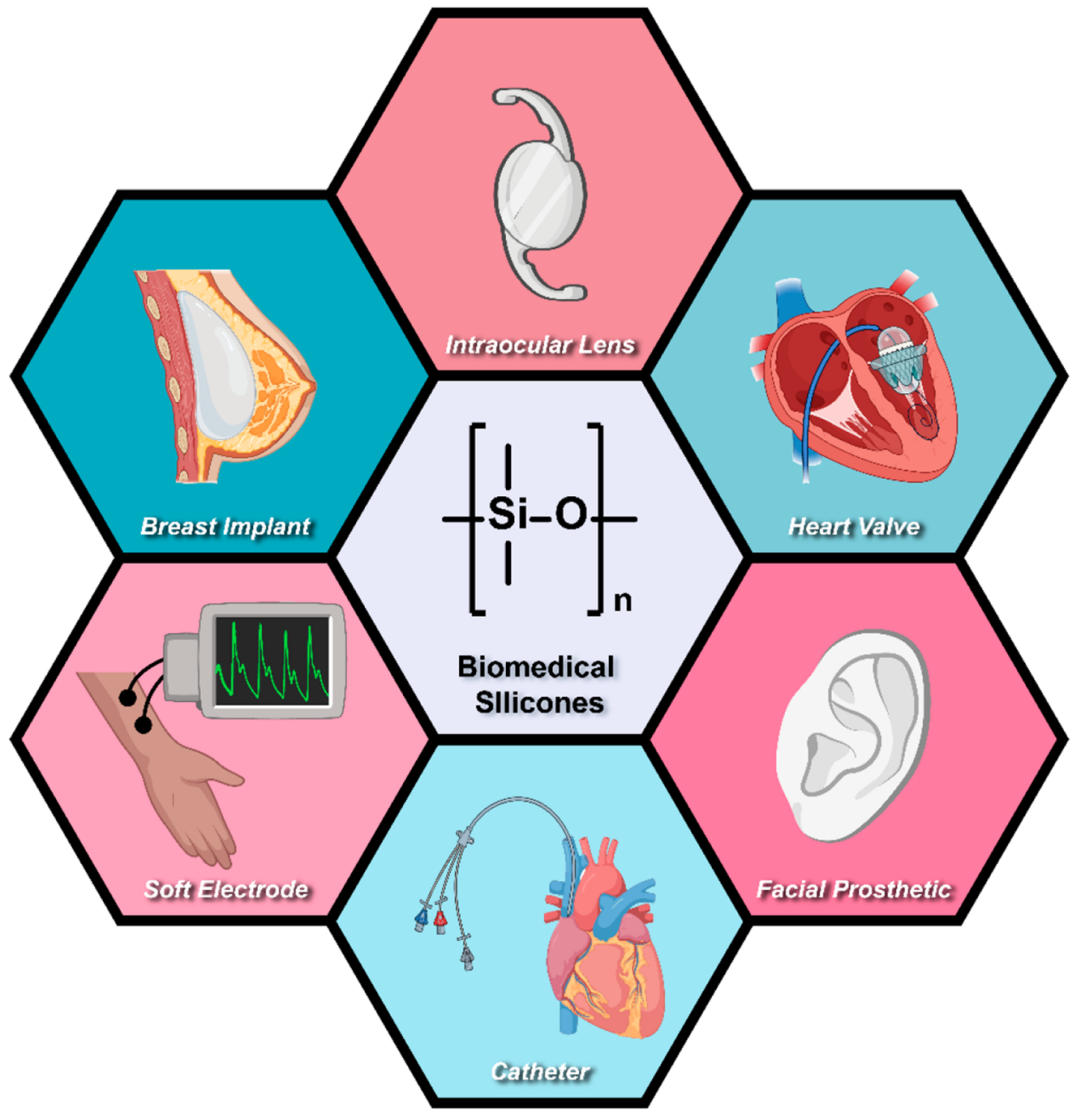
Pervasive utility of silicones for biomedical devices. Recent strategies based on modifications using additives can improve surface, rheological, mechanical, and electrical properties are described herein.

Silicones have a long history of use in biomedical devices, including cardiovascular (e.g., hemodialysis catheters and heart valve prostheses),^1^ ophthalmic (e.g., intraocular lenses [IOLs] and contact lenses),^2,3^ plastic and reconstructive prostheses (e.g., breast implants and facial prosthetics),^4,5^ and soft electrodes (e.g., electrocardiogram, electroencephalogram, and blood pressure;^6^Figure 1).

The utility of biomedical silicones is attributed to their unique properties stemming from the siloxane (Si–O–Si) backbone that features a high degree of flexibility and chemical stability.^7,8^ Upon cross-linking, the resulting silicones display unique elastomeric mechanical properties and resistance to degradation, as well as oxygen permeability. However, surface, rheological, mechanical, and electrical properties of silicones can limit their utility.^9^ Successful modification of silicones to address these limitations could lead to superior and new biomedical devices.

The last comprehensive review on the modification of biomedical silicones was published by Abbasi et al. over 20 years ago.^10^ Recent reviews either do not focus solely on silicones or only discuss one type of silicone modification strategy.^11−15^ Historically, several general strategies have been considered to modify properties of silicones. First, the pendant group chemistry may be altered from the dimethyl groups of polydimethylsiloxane (PDMS), the most widespread type of silicone. For instance, diphenyl silicones exhibit improved optical properties and thermal stability.^16^ Second, cross-linking density, afforded by terminal or pendant reactive groups, can also be used to tailor mechanical properties.^17^ In another broadly used strategy, silica fillers are added to reinforce silicones for improved strength and toughness.^18^ More recently, silicones have been combined with other polymers, such as in the case of interpenetrating polymer networks (IPNs).^19−21^ This strategy is exemplified in the creation of silicone hydrogel contact lenses, which are IPNs comprised of a dimethyl silicone and a thermoplastic, hydrophilic polymer.^22^ Thus, the oxygen permeability of the silicone and lubricity stemming from the hydrated hydrophilic polymer are synergistically combined.^22^ More recently, additives have been leveraged to modify biomedical silicones and are highlighted herein.

## 2 Overview of Silicone Cure Chemistries

**Figure 2:**
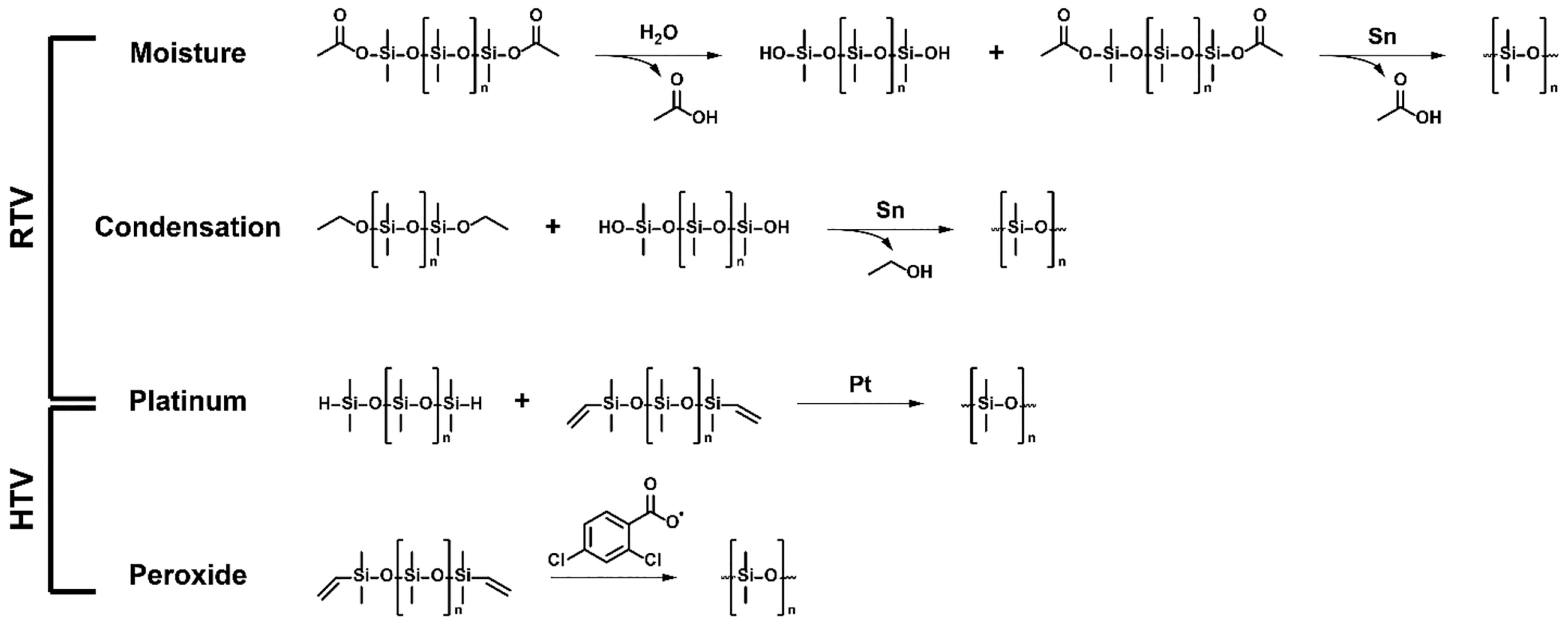
Silicone cure chemistries.

Silicones are formed via different curing chemistries that vary in terms of cross-linking group, catalyst type, and cure conditions that must be considered for biomedical device fabrication.^23^ Silicone cure systems are commonly broken down into two main categories, room-temperature-vulcanizing (RTV), and high-temperature-vulcanizing (HTV; Figure 2).^8^ RTV silicones include one-component moisture cure, and two-component condensation or addition cure systems. Moisture-cured silicones proceed through the hydrolysis of functional groups (e.g., acetoxy, alkoxy, and oxime) by moisture in the air, resulting in silanol (Si–OH) groups, which can then further cure through tin (Sn)-catalyzed condensation.^24^ The reaction side products, such as acetic acid (e.g., acetoxy cure), can take up to a week to evaporate from the cured silicone. Additionally, the rate of moisture penetration limits the thicknesses of moisture-cured silicones and so are generally used only in systems requiring thin films, coatings, or adhesives. Two-component condensation also relies on a Sn-catalyst but does not require atmospheric moisture and therefore can be used to prepare larger objects. The use of Sn-catalysts may cause potential toxicity for implanted devices, particularly if levels are not minimized.^25^ Moisture-cured silicones also suffer from shrinkage caused by the evaporation of condensation products and so can present challenges for devices that require precise tolerances. Additionally, cured silicones rely on a platinum (Pt)-catalyzed hydrosilylation reaction between silane (Si–H) and vinyl groups. Pt has shown to be nontoxic in its zero-oxidation state,^4^ and is also commonly used among HTV silicones for accelerating curing. HTV silicones also include peroxide cure systems that rely on the free radical polymerization of vinyl groups. Peroxide catalysts can lead to the formation of voids caused by volatile byproducts.^26^ Care must also be taken to remove these byproducts post curing to avoid toxicity issues.^27^

## 3 Surface Modifications

**Figure 3:**
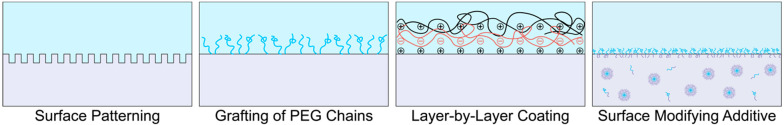
Surface modification of silicones and aqueous interface behavior.

Silicones surfaces are characterized by low surface tension and hydrophobicity.^28^ This is attributed to the low intermolecular forces of nonpolar pendant groups (e.g., methyl) and their often compact size that obscures the polar contributions of the siloxane backbone. This hydrophobicity renders silicones highly susceptible to biological adhesion.^29−34^ Adhesion is mediated by nonspecific protein adsorption wherein a conformational change orients the protein’s hydrophobic domains to the silicone surface and hydrophilic domains to the aqueous surrounding of the body. The decrease in silicone surface energy caused by protein adsorption exceeds the entropic loss caused by the conformational changes, thus making adsorption thermodynamically advantageous.^35^ Protein adsorption initiates the foreign body reaction (FBR),^36,37^ as well as thrombosis and infection,^38−40^ and can lead to eventual device failure.^41−44^ Thus, numerous chemical and physical approaches have been explored to modify the surfaces of silicones, particularly focusing on direct surface treatments to induce hydrophilicity.^45−51^ A primary obstacle is the susceptibility of modified silicone surfaces to hydrophobic recovery, stemming from the unique chain flexibility and mobility of the siloxane backbone.^52,53^ For instance, ionized gas (e.g., oxygen plasma) can introduce polar hydroxyl groups to the silicone surface, but are retained only if immediately immersed and maintained in an aqueous solution.^54−57^ Thus, any successful surface modification must contend with this reorganization mechanism to ensure long-term stability. For the surface modification of silicones, recent approaches to reduce biofouling have focused on surface patterning, surface grafting, layer-by-layer (LBL) coatings, and blending with surface modifying additives (SMAs; Figure 3).

Surface patterning relies on the use of macro-, micro-, and nanotopographies to induce changes in surface thermodynamic interactions. Patterning can either enhance or discourage biofouling based on pattern length scale, height, and feature spacing.^58^ Silicone IOL haptics^59,60^ and textured breast implants^61^ have utilized patterning to promote cell and eventual tissue growth in order to inhibit postsurgical movement. Haptics, the “arms” connected to the optic of an IOL, serve a 2-fold purpose of providing radial tension to the capsular bag and securing of the IOL. To improve long-term rotational stability, newer IOLs rely on “frosting” of the haptics via a surface pattern created during the molding process. The pattern increases frictional forces of the haptics with the capsular bag and allows for enhanced cell growth to secure the IOL.^62,63^ Similarly, breast implants have historically relied on texturing to inhibit implant movement^64−66^ and also to reduce the rate of capsular contracture.^67,68^ Doloff et al. (2021)^69^ showed that contracture was most reduced with patterns having smaller and more abundant roughness features. However, texturing can lead to negative results. Breast implants with macrotexture (e.g., Biocell) have shown higher rates of failure stemming from wear debris from the textured surface that led to chronic inflammation, pain, and their eventual recall.^70−72^

**Figure 4:**
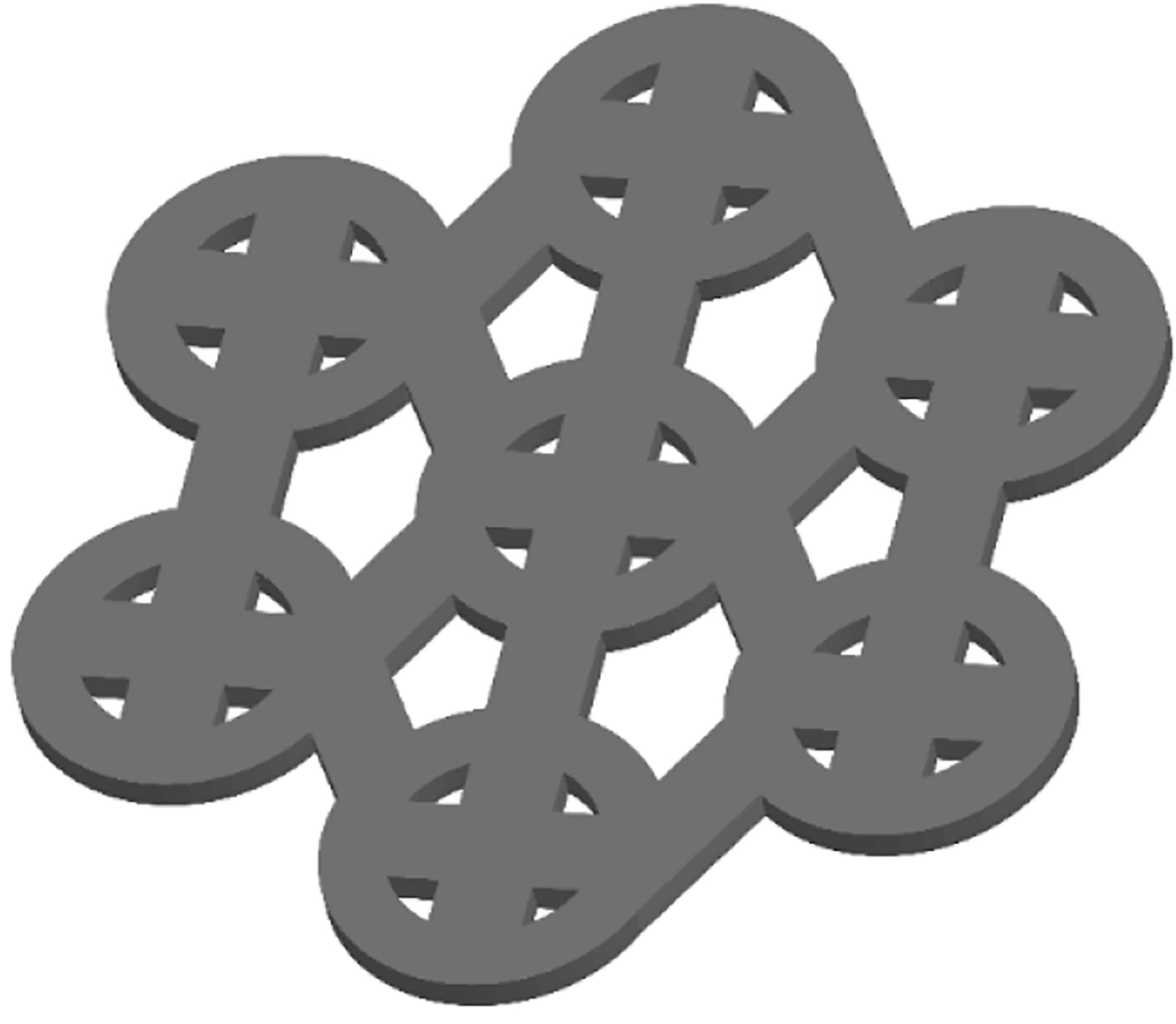
Representation of silicone surface pattern described by Atthi et al. (2022) for reduction of biofouling.

In other cases, patterning of silicones has been leveraged to reduce biological adhesion. However, silicones are highly susceptible to pattern deformation caused by external loads due to their characteristic low modulus. Atthi et al. (2022)^73^ designed a durable silicone micropattern with improved antifouling properties (Figure 4). The design relied on the Wenzel model of contact angle, wherein surface roughness factor, a ratio of surface area of the rough surface and surface area of the ideal surface, is maximized.^74^ Combining this approach with interconnected features allowed for a more robust pattern than the typical pillared approach. Unfortunately, the addition of complex surface patterning may not be feasible for many biomedical devices as it can be time-consuming and limited by device design parameters.

One of the primary approaches for hydrophilization of silicone surfaces is the grafting of polyethylene glycol (PEG).^75,76^ PEG is known for its exceptional protein resistance,^75−78^ and several mechanisms contribute to the efficacy of grafted chains. An excluded volume effect is induced by the flexibility and conformational mobility of the PEG backbone, resulting in steric repulsion of proteins and blocking of underlying adsorption sites.^79^ Grafted PEG chains also form a hydration layer which blocks interactions between proteins and the material surface, thereby eliminating protein conformational changes necessary for adsorption.^80^ The protein resistance of grafted PEG has shown great success on “ideal” substrates (e.g., glass, gold, and silicon).^78,81−85^ Yet, on polymer substrates, a decrease in effectiveness is observed due to issues with surface grafting density, low control of chain length, and disruption caused by shear forces.^86^ PEG brushes have been formed on silicone surfaces with a variety of chemistries, but relies on first pretreating the surface (e.g., oxygen plasma) or the use of a functionalized silicone (e.g., silanol and silane). In general, PEG-grafted silicone surfaces are observed to lose efficacy ∼30 days under flow and aqueous conditions.^87^ In addition to PEG, other hydrophilic polymers have been grafted onto silicone surfaces to induce hydrophilization. Zwitterionic groups such as sulfobetaines and carboxybetaines are commonly used due to their enhanced wettability caused by the polarity of the charged groups.^87−92^

LbL coatings have been formed on silicones to modify surfaces properties,^93^ with potential for superior stability versus grafted polymer chains.^94^ LBL coatings are comprised of alternating layers held together by secondary forces. Most commonly, LbL coatings are formed from alternating positively and negatively charged polymers applied sequentially by dipping.^95−97^ This allows for fine control of the coating thickness and complete surface coverage. Silicone surfaces are typically first treated with oxygen plasma followed by grafting of carboxylic acid functional groups to create a charged surface that can support formation of the LbL coating. Several studies have reported silicones with LbL coatings comprised of alternating layers of hyaluronic acid (HA) and a polycation (e.g., chitosan and poly-l-lysine).^98−100^ The resulting LbL-modified silicone surfaces displayed significant improvement in hydrophilicity as well as a decrease in cell adhesion. Unfortunately, charged layers of LbL coatings may be susceptible to rearrangement or delamination,^101^ as well as increase interactions with proteins.^102^ Vaterrodt et al. (2016)^103^ formed LbL coatings on silicones that incorporated zwitterionic polymers as well as peroxide-producing enzymes for antifouling and antibacterial properties, respectively. A freeze-drying step was utilized to improve immobilization of the enzyme and to reduce surface roughness. Overall, strategies exist to improve LBL stability and functionality. However, the associated complexity of chemistries and processing may prove to be an obstacle for translation to medical devices.

The use of SMAs to modify silicone surfaces is an appealing strategy given the relative simplicity of incorporation through blending. Typically, SMAs are composed of amphiphilic block copolymers wherein one portion of the chain is hydrophilic and the other is hydrophobic.^104−107^ Exposure to the aqueous, biological environment creates a thermodynamic incentive for the restructuring of the hydrophilic portions to the material surface. Meanwhile, the hydrophobic portions interact with the bulk material, ensuring proper dispersion and inhibiting leaching. Our research group has previously reported the modification of silicones, both a room temperature vulcanization (RTV) and addition cure system, with poly(ethylene oxide) (PEO)-silane amphiphiles (PEO-SAs). These were comprised of an oligo(dimethylsiloxane) (ODMS_*m*_) tether, a PEO headgroup (PEO_8_), and either a triethoxysilane (TES) group [α-(EtO)_3_Si-(CH_2_)_2_-ODMS_*m*_-*block*-PEO_8_-OCH_3_; *m* = 13 or 30] or a silane (Si–H) group [H-Si-ODMS_*m*_-*block*-PEO_8_-OCH_3_; *m* = 13 or 30]. The resulting modified silicones exhibited rapid and substantial water-driven surface hydrophilicity, resulting in broad spectrum biofouling resistance (e.g., proteins, bacteria, platelets, and lens epithelial cells).^53,108−117^ In contrast, silicones modified with a PEO-silane (i.e., no siloxane tether) did not exhibit such surface and antibiofouling behaviors. Thus, the siloxane tether is hypothesized to improve the miscibility of the amphiphilic SMA in the silicone bulk, improving the ability of PEO segments to migrate to the aqueous interface.

## 4 Rheological Modifications

**Figure 5:**
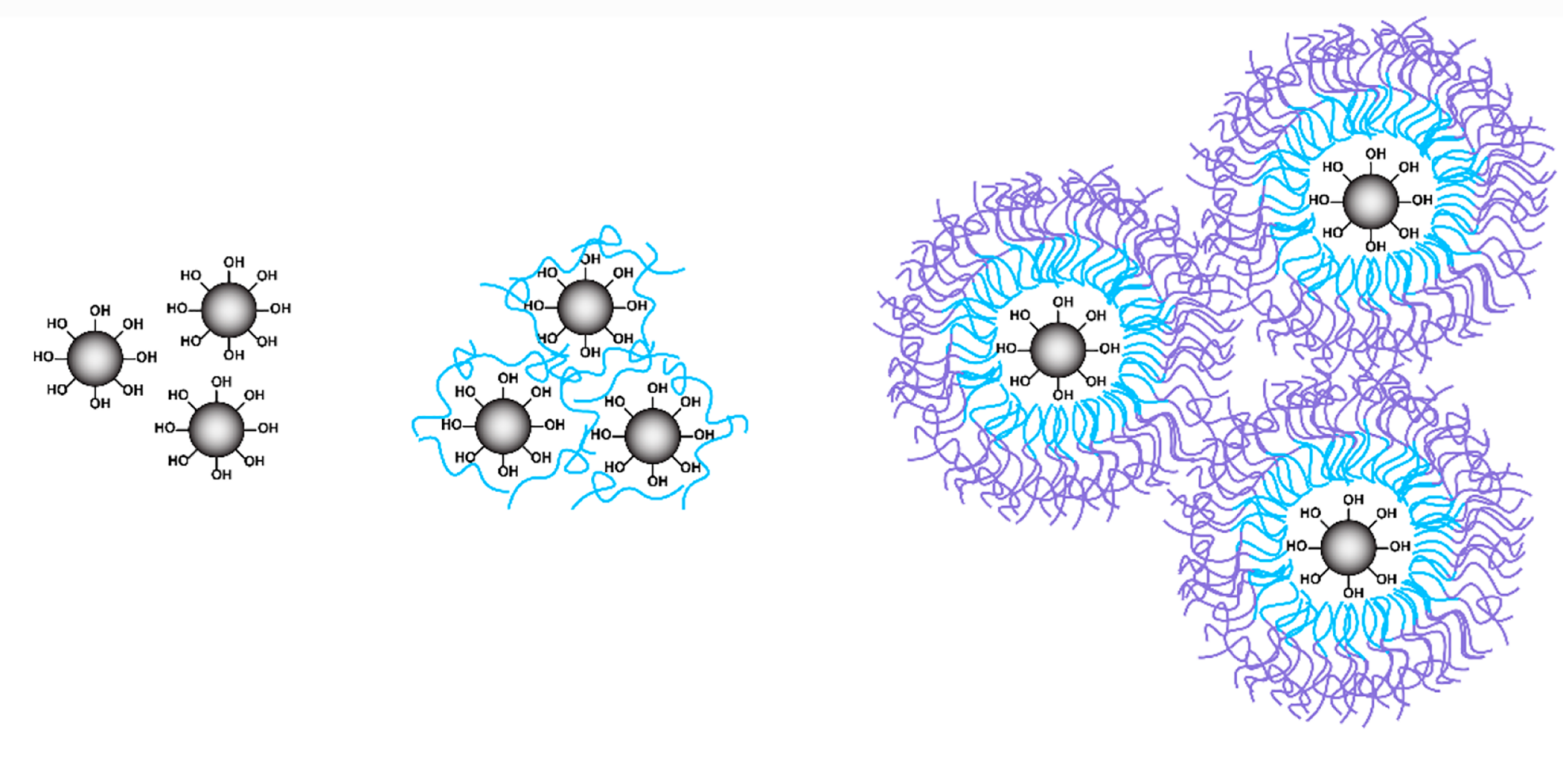
Rheological modification of silicone to create printable inks rely on silica fillers (left) and thixotropic additives (THXAs), such as PEG (middle) and amphiphiles (right).

Silicones are useful in many medical devices which require intricate structures, such as flexible electronics, soft robotics, and maxillofacial prostheses.^118−120^ Traditional fabrication techniques like compression molding, extrusion, and spin coating are limited by resolution, cost, and time.^121−123^ Direct ink write (DIW) 3D printing on the other hand, provides a more robust mechanism for the fabrication of silicone devices.^124^ This process requires that the “ink” exhibit thixotropic rheological behavior.^125^ In other words, the silicone ink must undergo fluidization at high shear and stiffening at low shear or rest.^126^ Thus, to improve printability of silicones, rheological modifiers such as silica filler, and thixotropic additives (THXAs) have been used (Figure 5).

To improve strength and toughness, silicones are often reinforced with silica fillers at levels of up to ∼30 wt %.^127^ The chemical similarity of silica fillers and silicones gives rise to their compatibility, facilitating dispersion.^128,129^ The silanol-containing surface of silica can also be refined with numerous chemistries to further enhance dispersion.^130,131^ Silica–silicone intermolecular interactions can give rise to thixotropy to form printable inks,^129^ with shearing of the silica–silicone network producing fluidification during extrusion.^125^ However, depending on the silicone, the requisite silica loading levels result in poor compatibility of the mixture and increased nozzle pressure during printing.^132^ In a study by Zhou et al. (2019),^124^ printable silicone inks were prepared by combining nonsurface modified silica filler (up to 8 wt %) with various commercial silicones. However, in another study, for Sylgard 184 (a silica-filled Pt-cure silicone), neither incorporation of ∼17 wt % hexamethyldisilazane (HMDS)-treated or dimethyldichlorosilane (DiMeDi)-treated silica alone was able to produce printable inks.^133^ Other common fillers such as aluminum oxide, titanium oxide, and graphite have been investigated for their use as thixotropic additives for silicones; however, they only achieved printability when silica filler was also added.^132^ Bai et al. (2020)^134^ used polytetrafluoroethylene (PTFE) micropowder as a substitute for silica. PTFE’s nonpolarity allowed for the creation of improved polymer–filler interaction and resulted in a thixotropic ink without the need for silica filler. However, to achieve the necessary properties for DIW inks, the PTFE had to be loaded at high amounts (55 wt %).

THXAs may decrease the required silica loading to form printable silicone inks, attributed to the increase in silica–matrix interactions. Courtial et al. (2019)^135^ reported that the incorporation of PEO in a silica-filled (0.5–8 wt %) silicone led to thixotropy through H-bonding with the silica’s silanol surface groups. PEO of lower molecular weights (450 g/mol) was able to improve rheological properties, without inducing a plasticizing effect in the final prints. However, a printable ink could not be achieved, attributed to weak PEO–matrix interactions. Our group further demonstrated that PEO was not an effective THXA for Sylgard 184.^133^ Thus, amphiphilic PEO-SAs (i.e., comprised of siloxane tethers and PEO segments) of varying architectures were evaluated as alternatives. Star and triblock PEO-SAs (5 wt %) were able to create printable inks for Sylgard 184 that also contained ∼17 wt % DiMeDi-silica filler. Formulations based on star PEO-SA produces surfaces were also capable of water-driven surface restructuring and so are anticipated to enhance antibiofouling behavior.

## 5 Electrical Modifications

**Figure 6:**
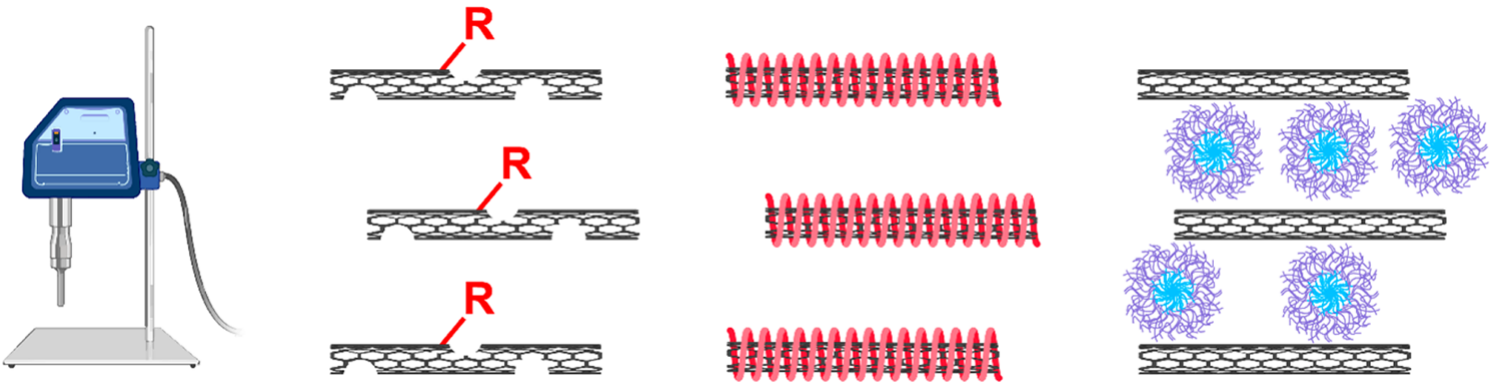
Methods for dispersing CNTS (left to right): sonication, covalent surface modification, polymer wrapping, and amphiphilic DSPAs.

The use of wearable continuous monitoring devices has been prompted by a reduction in the size of electronic components.^136^ Continuous monitoring has the advantage of alerting users to temporal changes in biological indicators (e.g., blood pressure, blood glucose, heart rate),^137−139^ rather than relying on intermittent measurements. This keeps users informed about important changes and trends that may otherwise be missed. Many such devices currently rely on light sensing which becomes less accurate with higher levels of pigmentation or subcutaneous fat.^140,141^ Thus, continuous monitoring shifted to electrical sensing, which relies on hard metal electrodes (e.g., Ag/AgCl). However, these lack the ability to conform to the skins surface and increase noise, leading to poor signal quality.^142^ Soft, gel-based, Ag/AgCl electrodes allow for a conformal fit, but are limited by skin irritation and limited long-term stability.^143^ This prompted the demand for flexible, dry skin electrodes. Silicones’ low modulus and elastomeric nature make them ideal candidates to interface with skin.^144^ To achieve the necessary electrical properties, silicones may be loaded with conductive fillers such as carbon black, graphene, and carbon nanotubes (CNTs).^145−148^ Silicone-based CNT composites hold particular promise,^149,150^ as the high aspect ratio of CNTs allows them to reach a theoretical percolation threshold at relatively lower loading levels.^151−153^ However, CNTs suffer from strong van der Waals interactions, making their dispersion in polymer matrices difficult.^154^ Current techniques for improving their dispersion include mechanical separation, surface modification, polymer wrapping, and the addition of dispersive additives (DSPAs), such as surfactants (Figure 6).

Mechanical separation relies on the use of high shear mixing or sonification to interrupt the van der Waals interactions of CNTs. These methods can cause damage to the tubes as the energy imparted into the mixture is not specifically targeted.^142,149^ This decreases their aspect ratio and, in some cases, reduces their ability to readily transport electrons, thereby necessitating higher loading amounts. Furthermore, mixing times can be upward of 15 h and have specific viscosity requirements, making broad adoption difficult. Chemical surface modifications instead decrease aggregation by directly interrupting tube–tube interactions.^155^ This is commonly achieved through the addition of carboxyl groups to the CNT surface. Yet, these modifications also lead to a decrease in intrinsic conductivity due to fracturing of CNTs, thereby increasing the effective loading amount required to impart electrical conductivity.^142,149,156^

To avoid the damage incurred by the aforementioned dispersion techniques, other research has looked into the use of noncovalent modifications such as polymer wrapping. Polymer wrapping works similarly to surface modification; however, it relies on strong physical interactions rather than covalent linkages.^157−160^ Bai et al. (2017)^161^ achieved this through the use of polymethylphenylsiloxane (PMPS), which adsorbs onto the CNT surface through π–π stacking and methyl−π interactions. Unfortunately, polymer wrapping requires a precise balance between polymer desorption and adsorption. If there is too much adsorption, there are no tube–tube interactions, thereby eliminating the materials conductivity, and if there is too little, then separation does not occur. Dynamic dispersion using surfactants is instead used to avoid the pitfalls of polymer wrapping and other static dispersion methods.

Surfactants are characterized by their ability to form supramolecular structures (i.e., micelles, bilayers), caused by the thermodynamically favorable separation of their hydrophilic and hydrophobic moieties.^162,163^ These structures are leveraged to assist in the separation of carbon nanotubes by creating physical barriers between CNTs.^164^ Yang et al. (2020)^165^ used sodium dodecyl sulfate (SDS) in order to improve CNT separation in a silicone. Nonetheless, this required a pretreatment to impart negative charges at the surface of the CNTs to ensure proper adhesion with the positively charged SDS. Our group has focused on the creation and use of a PEO-silane DSPA, which would allow for simple CNT separation.^166^ DSPA architecture (linear and star) and siloxane length were systematically varied to investigate their impact on CNT dispersion. These were combined with CNTs and an addition cure silicone, without modification to CNTs, addition of solvents, or exhaustive mixing protocols. Silicone-CNT composites formed with PEO-SAs containing siloxane tethers with 12 repeat units achieved the highest conductivity (σ_DC_). The top-performing composite displayed a σ_DC_ ∼ 140× higher than that of a composite prepared with no PEO-SA. The skin-electrode impedance for the top performing composite achieved similar results versus an Ag/AgCl electrode. Thus, PEO-SAs may act as effective DSPAs for the convenient and effective formation of silicone–CNT composites for soft skin electrodes useful for long-term monitoring.

## 6 Conclusion

In summary, numerous additive-based approaches have recently been leveraged to improve the outcomes of silicones used in biomedical applications. These ideally seek to modify silicones to permit tailoring of various properties key to success. Surface modifications selectively tune protein adsorption to improve device longevity by reducing failure caused biofouling. Rheological modifications enhance the use silicones for 3D printing applications, expanding the complexity of device designs. Finally, electrical modifications permit silicones to be used for advanced healthcare sensing. Given the potential impact on medical devices, continued work to improve biomedical silicones is a critical endeavor.
